# What is the role of lipids in prion conversion and disease?

**DOI:** 10.3389/fnmol.2022.1032541

**Published:** 2023-01-10

**Authors:** Cyntia Alves Conceição, Gabriela Assis de Lemos, Caroline Augusto Barros, Tuane C. R. G. Vieira

**Affiliations:** ^1^Institute of Medical Biochemistry Leopoldo de Meis, Federal University of Rio de Janeiro, Rio de Janeiro, Brazil; ^2^National Institute of Science and Technology for Structural Biology and Bioimaging, Federal University of Rio de Janeiro, Rio de Janeiro, Brazil

**Keywords:** prion diseases, prion protein, protein-lipid interaction, aggregation, neurodegenerative disease

## Abstract

The molecular cause of transmissible spongiform encephalopathies (TSEs) involves the conversion of the cellular prion protein (PrP^C^) into its pathogenic form, called prion scrapie (PrP^Sc^), which is prone to the formation of amorphous and amyloid aggregates found in TSE patients. Although the mechanisms of conversion of PrP^C^ into PrP^Sc^ are not entirely understood, two key points are currently accepted: (i) PrP^Sc^ acts as a seed for the recruitment of native PrP^C^, inducing the latter’s conversion to PrP^Sc^; and (ii) other biomolecules, such as DNA, RNA, or lipids, can act as cofactors, mediating the conversion from PrP^C^ to PrP^Sc^. Interestingly, PrP^C^ is anchored by a glycosylphosphatidylinositol molecule in the outer cell membrane. Therefore, interactions with lipid membranes or alterations in the membranes themselves have been widely investigated as possible factors for conversion. Alone or in combination with RNA molecules, lipids can induce the formation of PrP *in vitro*-produced aggregates capable of infecting animal models. Here, we discuss the role of lipids in prion conversion and infectivity, highlighting the structural and cytotoxic aspects of lipid-prion interactions. Strikingly, disorders like Alzheimer’s and Parkinson’s disease also seem to be caused by changes in protein structure and share pathogenic mechanisms with TSEs. Thus, we posit that comprehending the process of PrP conversion is relevant to understanding critical events involved in a variety of neurodegenerative disorders and will contribute to developing future therapeutic strategies for these devastating conditions.

## 1. Introduction

Transmissible spongiform encephalopathies (TSEs), also known as prion diseases, are a group of fatal neurodegenerative disorders that directly affect the central nervous system (CNS), causing loss of neuronal cells and, consequently, neurological symptoms (Greenlee and Greenlee, 2015; Asher and Gregori, 2018). The TSEs that affect humans are Creutzfeldt-Jakob disease (CJD), fatal familial insomnia (FFI), kuru, Gerstmann-Sträussler-Scheinker (GSS) syndrome, and variably protease-sensitive prionopathy (VPSPr; Collins et al., 2004; Gambetti et al., 2011; Baldwin and Correll, 2019; Liberski et al., 2019). The structural conversion and accumulation of prion protein (PrP) play a significant role in the development of TSEs (Gill and Castle, 2018).

The cellular prion protein (PrP^C^) is encoded by PRNP, a highly conserved gene in mammals located on chromosome 20 in humans (Sarnataro et al., 2017). PRNP encodes a sequence of 253 amino acids that undergo post-translational modifications in the endoplasmic reticulum. These modifications include the removal of a signal peptide in the N-terminal domain (1-22) that directs PrP^C^ to the plasma membrane; the removal of a signal peptide in the C-terminal domain (232-253) in the endoplasmic reticulum, and the attachment of a glycosylphosphatidylinositol (GPI) anchor; addition of glycans (N-181 and N-197), and the formation of disulfide bonds (C-179 and C-214; Stahl et al., 1987; Haraguchi et al., 1989; Harris, 2003). The GPI anchor is added to PrP^C^ *via* the GPI-transamidase enzyme (Puig et al., 2014; Sarnataro et al., 2017). PrP^C^ is widely found in CNS cells and is also expressed in other tissues, such as the heart and lungs (Bendheim et al., 1992; Wulf et al., 2017; Gill and Castle, 2018); it is found in regions of lipid rafts anchored by the GPI in the extracellular membrane (Prado et al., 2004).

The mature prion protein structure (PrP23-231) can be structurally divided into N- and C-terminal domains. The N-terminal domain is intrinsically disordered, with a repeated octapeptide sequence (PHGGGWGQ), containing histidine residues that are important for interaction with metallic ligands such as copper (II) (Brown et al., 1997; Salzano et al., 2019). The C-terminal domain is structured and globular, with three α-helices and a small antiparallel β-sheet (Heske et al., 2004; Acevedo-Morantes and Wille, 2014).

PrP^C^ plays many different roles in cells since it interacts with many other partners. These functions are related to metal ion metabolism, neurotransmission, neurogenesis, neuroprotection by acting as an antioxidant, cell–cell adhesion, and memory, among others (Das and Zou, 2016; Linden, 2017; Wulf et al., 2017). The cellular location of PrP^C^ at the plasma membrane may be related to its cell signaling function, as discussed below. PrP^C^ may interact with membrane lipids and associate with other transmembrane proteins, thereby transmitting signals into the intracellular compartment (Legname, 2017; Sarnataro et al., 2017).

Conformational changes in PrP^C^ cause TSEs; it has its structure rich in α-helices (about 40%) and a small percentage of β-sheets (about 3%), which transforms into a structure enriched in β-sheets (about 45% β-sheets and 30% α-helices) called prion scrapie (PrP^Sc^; Pan et al., 1993; Prusiner, 1998; Wulf et al., 2017). The conversion of PrP^C^ to PrP^Sc^ leads to biochemical changes in the physicochemical properties, increasing the tendency to aggregation, resistance to protease digestion, and partial resistance to heat and denaturing agents (Prusiner, 1998; van Rheede et al., 2003). The diseases caused by PrP^Sc^ can have an infectious and genetic origin and have been classified as hereditary, acquired, or sporadic. Mutations in the PRNP gene are associated with the hereditary form, while there is a spontaneous conversion of PrP^C^ to PrP^Sc^ in the sporadic form. In the acquired form, transmission can occur in several ways, such as using surgical instruments and ingesting contaminated food (Will, 2003; Geschwind, 2015).

Explanations still need to be made available for how exactly PrP^C^ structural changes initiate and propagate in the misfolded form. According to the protein-only hypothesis, the presence of PrP^Sc^ alone is sufficient to induce PrP^C^ conversion, as the former acts as a template, recruits PrP^C^, and causes conformational changes for more PrP^Sc^ formation and subsequent aggregation (Prusiner, 1998; Baskakov and Bocharova, 2005). Some studies reproduced this conversion hypothesis *in vitro* but with low efficiency (Deleault et al., 2005; Saá et al., 2006; Zhang et al., 2013). *In vitro* conversion in the presence of other molecules was more efficient (Deleault et al., 2003; Geoghegan et al., 2007; Kovachev et al., 2019). Therefore, it is hypothesized that the presence of cofactors benefits PrP conversion (Cohen and Prusiner, 1998; Silva et al., 2010a; Wang et al., 2010a,b). Other biomolecules, like lipids, RNA, DNA, and other proteins at the cell membrane and in the cytoplasm, could act as cofactors that accelerate prion structural conversion and subsequently modulate infectivity and toxicity.

In this review, we provide information about membrane lipid interaction with prion protein and the role of this interaction in PrP function and the conversion process. First, we discuss prion protein attachment to cell membranes and the effects of this interaction on prion structure and stability. Then, we discuss the roles of PrP-lipid interactions in physiology. Finally, we relate studies that investigate lipid involvement in RNA- and lipid-mediated PrP conversion and the toxic effects of this process.

## 2. Prion association with lipids in physiology

### 2.1. Membrane lipids and their importance

Cells and some cell organelles are delimited by a lipid membrane organized in bilayers of two lamellae, where the hydrophobic portion of the lipids hides from the water, and the hydrophilic portion interacts with the outer and inner cellular spaces. This lipid bilayer is responsible for cell protection and cell–cell communication and selectively internalizes some molecules to the cytoplasm (Simons and Sampaio, 2011). A vast repertoire of lipid species can participate in the structure of membrane bilayers. Most lipids in the mammalian cell membrane are glycerophospholipids (GLPs), sphingolipids (SPs), and cholesterol. GLPs, the major lipid components of the membranes, comprise a glycerol backbone linked to a hydrophobic portion of two acyl chains and a hydrophilic headgroup with phosphoric acid. This basic structure of a GLP is named phosphatidic acid (PA). The interaction of the PA headgroup with alcohol molecules leads to the formation of a diversity of GLPs, namely phosphatidylinositol (PI), phosphatidylethanolamine (PE), phosphatidylcholine (PC), and other GLPs. SPs comprise a sphingosine backbone linked to an acyl chain on its amine group, and a headgroup, such as phosphocholine, phosphoethanolamine and others, on its hydroxyl group.

The sphingosine backbone also forms the core of glycolipids, which may interact with lipid bilayers. In the case of glycolipids, the hydroxyl group of the sphingosine binds to glycan units (Harayama and Riezman, 2018). Each cell organelle has different membrane lipid distribution, influencing its fluidity and function. High amounts of cholesterol contribute to the formation of less impermeable bilayers; for instance, this lipid is found in higher proportions in the plasma membrane (PM), which is essential for controlling molecule exchanges with the external environment. Cholesterol and SPs are associated with more organized regions of the membrane bilayers, such as caveolae and lipid rafts (Simons and Sampaio, 2011; Egawa et al., 2016).

The lipid bilayer also comprises transmembrane proteins and proteins that interact with the membrane through glycolipids, allowing communication between cells and activating various physiological mechanisms (Egawa et al., 2016). PrP^C^ is attached to the outer leaflet of cellular membranes through a GPI anchor, bound to lipid raft regions *via* the amino acid residue S231 (Rudd et al., 2001; Taylor and Hooper, 2006). GPI is composed of a core made of three mannose and one glucosamine residue. A PI headgroup with a saturated acyl chain connects to the GPI glucosamine residue, and during the traffic to the cell membrane, sialic acid can be linked to one of the mannose residues (Taylor and Hooper, 2006). Lipid rafts are lipid membrane microdomains formed by the ordered assembling of cholesterol and SPs that create a liquid domain resistant to detergent solubilization, unsaturated GLPs, and proteins (Egawa et al., 2016). Changes in GPI composition and PrP^C^ attachment to lipid raft influence PrP’s physiological and pathological roles (Bate et al., 2016a,b).

PrP^C^ internalization is required for it to be trafficked to the secretory and recycling pathways. Two principal mechanisms of PrP internalization have been described: caveolae-dependent and clathrin-coated pit internalization (Shyng et al., 1994; Peters et al., 2003; Fehlinger et al., 2017). Caveolae are membrane invagination regions, considered a special class of rafts, which are rich in cholesterol and glycosphingolipids, and are coated by the protein caveolin-1; Caveolin-1 mediates signal transduction mechanisms and other physiological processes (Anderson, 1998; Peters et al., 2003; Echarri et al., 2007). PrP^C^ was found in the caveolae regions of the PM and trans-Golgi network (Peters et al., 2003). It was found that the depletion of cholesterol from these regions is associated with the impairment of PrP^C^ endocytosis, suggesting that the lipid is of great importance for caveolae-mediated PrP internalization and that PrP must be present in lipid raft-like domains to be internalized *via* caveolae (Marella et al., 2002; Bate et al., 2004; Cashion et al., 2022). Nonetheless, some neuronal cells do not have the machinery for caveolae internalization of proteins. Thus, other internalization pathways, like clathrin-mediated endocytosis, are important in these cells’ PrP internalization mechanism.

In contrast to the caveolae mechanism, PrP internalization mediated by clathrin-coated pits occurs out of the lipid rafts in non-raft membrane domains (Taylor and Hooper, 2006). Since the clathrin pathway is only related to the endocytosis of transmembrane proteins (Trowbridge, 1991), PrP internalization mediated by clathrin depends on its interaction with a transmembrane adaptor (Shyng et al., 1994, 1995; Taylor and Hooper, 2006). Moreover, deletions of N-terminal amino acid residues, or point mutations in its polybasic region, impair PrP endocytosis (Nunziante et al., 2003; Sunyach et al., 2003). Cu^2+^ binding to the octarepeat region at the N-terminal destabilizes the PrP structure, facilitating its translocation from lipid rafts (Taylor et al., 2005). Thus, the PrP N-terminal domain and the lipid membrane composition, together with anchoring through GPI, are critical for PrP endocytosis.

During endocytosis processing, PrP loses its stability and may detach from the GPI anchor, leading to the conversion and loss of its physiological functions (Chesebro et al., 2005; Bate et al., 2016c). Moreover, PrP^C^ localization in lipid rafts and the maturation or degradation process facilitate its interaction with various molecules in the membrane traffic. These interactions influence its physiological effects and the conversion process to PrP^Sc^.

### 2.2. The physiological role of PrP-lipid interaction

**Figure 1:**
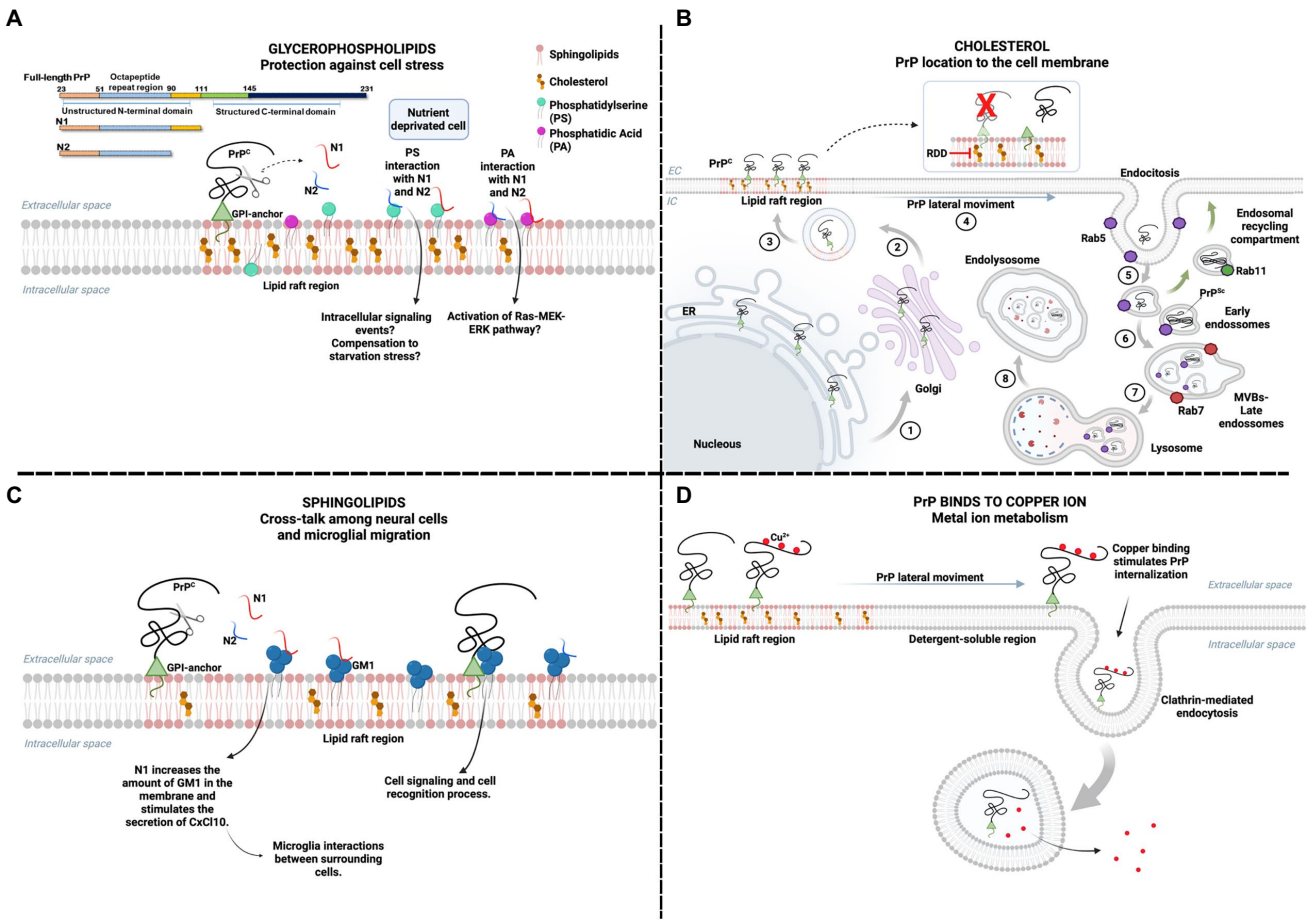
The physiological effects of PrP interaction with membrane lipids. Location on lipid rafts enables PrP^C^ interaction with various ligands, including membrane lipids. **(A)** Cellular starvation can alter the plasma membrane order, leading to PS exposure to the extracellular medium and increased PA. The interaction of N1 and N2 fragments, peptides derived from the proteolytic cleavage of PrP, may have a regulatory effect on cellular stress by binding to PS and PA. The interaction of N1 and N2 with PA can activate the Ras–MEK–ERK pathway and promote cell survival. At the same time, the interaction with PS can also activate pathways related to the same function. **(B)** PrP^C^ is formed in the endoplasmic reticulum, which undergoes post-translational modifications, such as binding to the GPI anchor. Subsequently, it is taken to the Golgi complex (1) and forwarded to the plasma membrane (2), mainly in lipid rafts (3). The importance of cholesterol in lipid rafts for the physiological location of PrP^C^ has already been reported. The process of internalization of PrP requires a lateral movement (4) to areas where the membrane is more soluble, outside the lipid rafts. Endosome motility is related to the function of Rab proteins, such as Rab 5 for early endosomes (5), Rab 7 for the multivesicular body (MVB)/late endosomes (6), and Rab11 for the endosomal recycling compartment (ERC) (green arrows). Conversion of PrP^C^ to PrP^Sc^ can occur within ERC and MVBs. MVBs fuse to lysosomes (7) and proteins are degraded in endolysosomes (8). The cleavage of PrP^C^ from the GPI anchor may favor PrP-lipid interaction and PrP conversion, but cells expressing PrP without GPI are not infected. Drugs that sequester cholesterol are raft dissociation drugs (RDD), known to inhibit PrP^Sc^ formation. **(C)** The interaction of GM1 ganglioside and N1 fragment promotes the increase of GM1 in the plasma membrane. It stimulates the secretion of the cytokine CxCl10, enabling the interaction between microglia and other surrounding cells. The interaction between PrP^C^ and GM1 has been reported, but the mechanisms triggered by this interaction have not been elucidated but are possibly related to cell signaling and cell recognition process. **(D)** PrP^C^ binds to copper ions and is involved in ion metabolism. It has already been reported that the interaction between copper and PrP^C^ stimulates the endocytosis of PrP. In the presence of copper, PrP^C^ leaves the lipid raft region and internalizes mainly *via* clathrin-mediated endocytosis, balancing the amount of copper inside and outside the cell. Created with BioRender.com.

Although the precise function of PrP^C^ remains elusive, its physiological participation in multiple cell-signaling pathways may be determined by its interaction with different lipids in the cell membrane (Figure 1; Godsave et al., 2015; Wulf et al., 2017). Structural and functional studies of PrP have revealed the presence of multiple membrane interaction motifs. At the N-terminus, an unstructured basic-hydrophobic region is predicted to be membrane-interactive since it inserts into micelles and may have a role in cell penetration (Magzoub et al., 2006). Multiple tryptophan residues in the octapeptide repeats between residues 60 and 91 interact with dodecyl phosphocholine (DPC) micelles regardless of the presence or absence of Cu^2+^ and insert into membranes (Dong et al., 2007). The protonation of the histidine residues in these repeats within low pH environments of endocytic compartments could favor interactions with acidic lipids (Morillas et al., 1999).

These membrane-interacting elements are disordered regions and may mediate pH-dependent associations with acidic phospholipid bilayers *via* relatively non-stereospecific electrostatic and hydrophobic interactions. The investigation of the interaction between full-length PrP and Supported lipid bilayers, a membrane mimetic surface, by single-molecule force spectroscopy revealed three lipid-binding regions at the PrP N-terminal: PrP^95-110^; PrP^23-51^ and the octapeptide repeat PrP^51-90^ (Pan et al., 2019). Motifs in the C-terminal domain of PrP, which shows structured elements, may mediate more specific lipid interactions (Overduin et al., 2021). Three proximal membrane interacting motifs, including V^122^GGL, which precedes the β1 strand, Y^169^SN between β2 and α2, and Y^225^YQR in α3, were implicated in the association with lipids, forming a single continuous binding surface with lipid membranes (Overduin et al., 2021).

The N-terminal fragments resulting from the physiological post-translational endoproteolytic cleavage of PrP^C^ – the N1 and N2 fragments – were shown to bind *in vitro* to phosphatidylserine (PS) and PA, which are components of cell membranes responsive to cellular stress. In a model of serum deprivation, the N2 fragment protected neural cells from disturbance in the cellular lipid environment, including externalization of membrane PS and increased PA levels (Haigh et al., 2015). This suggests that PrP’s protective role in cellular stress conditions may involve interaction with PS and PA (Figure 1A).

The N1 fragment, which also interacts with PS and PA, was shown to enhance cell viability in a co-culture of neuronal and microglia cells and, significantly, to modulate the interaction and cross-talk among the cells through an increase of the sphingolipid GM1 at sites of interaction in the membrane of microglial cells (Carroll et al., 2020; Figure 1C). GM1 is a ganglioside that resides in cholesterol-rich domains of the cell membrane, whose disruption has been related to the reduced migratory capacity of microglial cells (Kuipers et al., 2006), and PrP was shown to bind to GM1 directly (Sanghera et al., 2011). Since PrP is necessary for inducing cell migration by microglial stress-inducible protein 1 (STI1; da Fonseca et al., 2012), also a PrP ligand, the impairment in the migration of microglia by the disruption of cholesterol-rich domains may be related to the loss of interaction of PrP with GM1.

The physiological interaction between gangliosides and PrP was observed in neural and immune system cells like T lymphocytes. In human T cells, PrP was shown by immunoprecipitation to interact with the ganglioside GM3 specifically, the main component of glycosphingolipid-enriched microdomains (GEM) in the cell membrane (Mattei et al., 2002, 2004), which is involved in T-cell activation signaling by assembly with signal transducer molecules, such as Fyn and phosphorylated ZAP-70 (P-ZAP-70). Since PrP interacts with GM3, Fyn, and P-ZAP-70, it is proposed to be a component of the multimolecular signaling complex involved in ligand-specific T-cell activation, suggesting a role for PrP in this context. In a human T-cell line, PrP colocalized with GM1 and CD3, also components of the multimolecular T-cell receptor (TCR) complex, in response to hypothermic stress, which led to lymphocyte activation (Wurm et al., 2004). The depletion of cholesterol by methyl-β-cyclodextrin, which interferes with the interaction of GPI-anchored proteins such as PrP, impaired the hypothermal activation of T-cells (Wurm et al., 2004), reinforcing a role for PrP.

Cholesterol, also abundant in lipid rafts domains, was essential to the cell membrane localization of PrP in neurons since inhibiting cholesterol synthesis led to an accumulation of PrP in the Golgi compartment (Gilch et al., 2006). Therefore, cholesterol determines PrP roles depending on its cell membrane location (Figure 1B).

Nonetheless, the characteristic structural assemblies between lipids and proteins in lipid rafts are not confined to the plasma membrane. Raft-like microdomains are found in membranes of subcellular compartments like the endoplasmic reticulum, Golgi, and mitochondria (Hayashi and Su, 2003; Garofalo et al., 2005). Mitochondrial lipid raft-like microdomains have been proposed to regulate cell apoptosis in different cell types (Garofalo et al., 2015). Interestingly, PrP is present in the inner mitochondrial membrane of healthy brain tissue, suggesting a role for PrP in mitochondrial function (Faris et al., 2017). It is well known that mitochondria are an important modulator of cell apoptosis through the release of cytochrome C and the resulting activation of the caspase signaling pathway (Wang and Youle, 2009). PrP was also shown to modulate apoptosis in multiple conditions, exerting an anti-apoptotic role in neuronal and cancer cells (Kim et al., 2004; Roucou et al., 2005; Gao et al., 2019; Adhikari et al., 2021). Therefore, it is plausible to suggest that the anti-apoptotic role of PrP may involve its interaction with mitochondrial lipid raft-like microdomains.

Among the multiple roles of PrP in cellular homeostasis, its role as a copper-binding protein is the most well-accepted (Kawahara et al., 2021). Importantly, it may function as an antioxidant as it quenches free radicals generated by Cu^2+^ redox cycling (Haigh and Brown, 2006; Viles et al., 2008). The excess of free Cu^2+^ ions is toxic due to producing reactive oxygen species (ROS), such as superoxide and nitric oxide (Wong et al., 2008; Kodama et al., 2012). Studies show that enzyme superoxide dismutase (SOD) activity, crucial for controlling ROS homeostasis, is regulated by the presence of PrP^C^ in cell membranes (Brown et al., 1997, 1999; Sakudo et al., 2005). Brown et al. showed that adding Cu^2+^ to PrP induces SOD activity. This study compared the SOD activity of PrP^C^ extracted from wild-type and Prnp knockout mice (Prnp^-/-^) and showed that SOD activity is abolished in Prnp^-/-^ mice, suggesting that PrP^C^ is vital for SOD activity. Also, when Cu^2+^ was chelated in wild-type mice using diethyldithiocarbamate (DDC), PrP^C^-SOD activity was abolished (Brown et al., 1999).

The leading site for Cu^2+^ binding is the PrP^C^ N-terminal region, with four Cu^2+^ binding to four octarepeats (PHGGGWGQ), one to His96 and another to His111 (Sánchez-López et al., 2018). Binding to Cu^2+^ drives PrP^C^ lateral movement outside lipid rafts, stimulating endocytosis (Pauly and Harris, 1998; Taylor et al., 2005). It regulates Cu^2+^ levels and the activity of Cu-dependent enzymes (Figure 1D; Brown et al., 1997). It is also suggested to regulate PrP^C^ interaction with membrane partners, affecting PrP^C^’s physiological role (Posadas et al., 2022).

Alpha-cleavage, the main proteolytic event of PrP^C^, yields fragments N1, including residues 23-110, and C1, residues 111-231 with a free NH_2_-terminus, which remains membrane-bound and retains the copper binding site at His111 (Sánchez-López et al., 2018). Although it has only one coordination site for Cu^+2^ precisely at the alpha-cleavage site, PrP(111-115) peptide was proposed to bind Cu^2+^ depending on proton (pH) and copper-peptide ratios (Sánchez-López et al., 2018). Since the C1 fragment can represent up to 50% of total PrP^C^ at the PM (Altmeppen et al., 2012) and is exposed to fluctuations in copper concentration during synaptic transmission (D’Ambrosi and Rossi, 2015; Gromadzka et al., 2020), Cu^2+^ trafficking may be physiologically modulated by the membrane-bound C1 fragment.

Interestingly, a disturbance in lipid rafts composition by exposure to exogenous gangliosides GM1, GM3, and GD1a in cell culture, did not impact PrP cleavage and consequent generation of C1 and N1 fragments. However, it led to the structural rearrangement of PrP^C^ (Botto et al., 2014). In accordance, neither lipid raft location nor membrane anchorage of PrP^C^ was central for the generation of C1, since cells expressing (i) PrP-CTM, a PrP construct known to not localize in lipid rafts or (ii) a GPI-anchorless mutant PrP, produced a fragment analogous to C1 in cell lysates (Walmsley et al., 2009). However, the C1 fragment strongly colocalized with the lipid raft marker Cholerae Toxin B subunit, showing a preferential enrichment in raft regions (Botto et al., 2014), suggesting cholesterol and sphingolipids may be necessary for the Cu^2+^-binding property of this fragment, although in a manner not related to its alpha-cleavage.

## 3. Prion association with lipids in pathology

### 3.1. The importance of membrane environment for PrP conversion

PrP^C^ anchoring to lipid rafts was shown to increase its stability; thus, the absence of the GPI anchor may influence PrP susceptibility to conversion and aggregation (Figure 1B; Baron et al., 2002; Chu et al., 2014). Moreover, it has been reported that characteristics related to prion diseases, such as neuropathology and disease incubation time, are modified depending on the presence and composition of the GPI anchor (Chesebro et al., 2005; Bate et al., 2010; Bate and Williams, 2011). The absence of sialic acid in the GPI composition may change the lipid environment and be related to the reduction of PrP^Sc^ neurotoxicity (Bate and Williams, 2011; Bate et al., 2016d). A recent study showed that changes in the GPI signaling sequence of the PrP C-terminal domain generate a GPI anchor lacking sialic acid. The new composition was associated with increased prion disease incubation time and reduced PrP^Sc^ levels (Puig et al., 2019). Therefore, changes in the polysaccharide composition of the GPI anchor directly interfere with PrP conversion, becoming an attractive target to modulate PrP aggregation.

Cholesterol lipid is essential for PrP localization in lipid rafts; however, high concentrations of this lipid are cytotoxic as it leads to decreased membrane fluidity and membrane disruption, besides other toxic effects related to cholesterol oxidation. These changes may cause defects in integral membrane activity, cell signaling, and death (Tabas, 2002). Upon prion infection, enzymes involved in cholesterol synthesis are upregulated in neuronal cell lines and infected neurons (Bach et al., 2009). Also, cholesterol efflux from the brain to the circulation system is affected by reduced levels of the enzyme cholesterol 24-hydroxylase [Cytochrome P450 46A1 (CYP46A1)] in mice brains infected with PrP^Sc^ (Ali et al., 2021). Consequently, cholesterol levels are increased in neuronal cells and sequestered to membranes during prion disease progression, contributing to PrP^Sc^ pathogenic mechanism (Bate et al., 2008a,b,c; Cashion et al., 2022).

Inhibition of cholesterol synthesis using lovastatin and squalestatin reduced PrP^Sc^ formation in prion-infected cells (Taraboulos et al., 1995; Bate et al., 2004). The administration of efavirenz to N2a-infected cells, an allosteric activator of CYP46A1 enzyme, reduced PrP^Sc^ levels without affecting cell membrane stability nor PrP^C^ levels. Moreover, an increase in survival time and a decrease in the disease progression were observed in prion-infected mice treated with this drug (Taraboulos et al., 1995; Ali et al., 2021; Cashion et al., 2022). Drugs affecting lipid raft formation by biding cholesterol, such as filipin and amphotericin B, showed the same effect over PrP^Sc^ levels (Mangé et al., 2000; Marella et al., 2002). A drug affecting cholesterol transport named U18666A caused a redistribution of cholesterol from the plasma membrane to the intracellular space, reducing PrP^Sc^ in N2a cells, although it failed when administrated to infected mice (Klingenstein et al., 2006; Hagiwara et al., 2007). Thus, the stability of the PrP^C^ and its conversion must be significantly affected not only by the GPI anchor but also by the composition of the lipid raft itself. In this context, drugs that affect cholesterol metabolism are promising therapeutic candidates for prion diseases.

In addition to its importance in modulating lipid metabolism, the presence of PrP^C^ or its scrapie form is also essential for modulating endosomal trafficking processes and being modulated by it. PrP trafficking during the endocytic pathway favors the formation of resistant PrP (PrPres) in cell cultures infected with PrP^Sc^ (Caughey and Raymond, 1991; Marijanovic et al., 2009; Priola and McNally, 2009). Thus, PrP internalization is an essential step for the conversion into PrP^Sc^ and the associated aggregation.

PrP trafficking is essential for conversion and especially for prion transmission and propagation. The lateral translocation of PrP from the lipid raft region for clathrin-coated pits makes PrP more susceptible to conversion since the protein is less stable in non-raft regions (Taylor et al., 2005). Furthermore, once endocytosed, PrP traffics into early endosomes to be either directed to recycling and returns to the plasma membrane or to late endosomes to be degraded in lysosomes (Campana et al., 2005; Figure 1B). Studies suggest that PrP conversion occurs in the endosomal recycling compartment (Marijanovic et al., 2009) and in late endosomes (or multivesicular endosomes; Yim et al., 2015), which may propagate through secreted exosomes (Fevrier et al., 2004; Yim et al., 2015).

PrP^Sc^ alters Rab GTPases profile (Kovács et al., 2007; Shim et al., 2016), known to regulate intracellular transport and vesicle fusion, interfering with the endo-lysosomal pathway, and it may enhance conversion and toxicity. PrP^Sc^ infection also affects post-Golgi vesicle transportation of membrane proteins such as PrP^C^, which accumulates in the Golgi apparatus. Other membrane protein distributions are also affected by PrP^Sc^, such as insulin receptor, which is essential for neuroprotection, and attractin, which absence may have implications in spongiform degeneration since it plays a role in the myelination process. Thus, prion toxicity involves not only PrP^Sc^ activity but also the impairment of other membrane protein functions (Kuramoto et al., 2001; Barmada, 2005; Nelson and Alkon, 2005; Uchiyama et al., 2013). Moreover, PrP^Sc^ amyloid aggregates may accumulate on cell membranes, forming amyloid deposits. In neurons, PrP^Sc^ deposition is associated with dendritic degeneration in the early steps of prion disease, leading to severe synapses dysfunction (DeArmond and Bajsarowicz, 2010).

Other cells’ subcompartments may influence PrP aggregation (Campana et al., 2005). Genetic prion diseases, such as FFI and GSS, are characterized by point mutations in PrP amino acid sequence (Baldwin and Correll, 2019). Mutation in the PrP residue 117, associated with the GSS pathology, generates the accumulation of PrP with its C-terminal inside the ER lumen, which is associated with increased ER stress and PrP^Sc^ accumulation (Hegde et al., 1998). PrP is also found in the cytosol (Mironov et al., 2003; Levine et al., 2005), and the overexpression of PrP without its signal sequence for GPI interaction is associated with the formation of PK-resistant cytosolic aggregates (Godsave et al., 2015).

**Figure 2:**
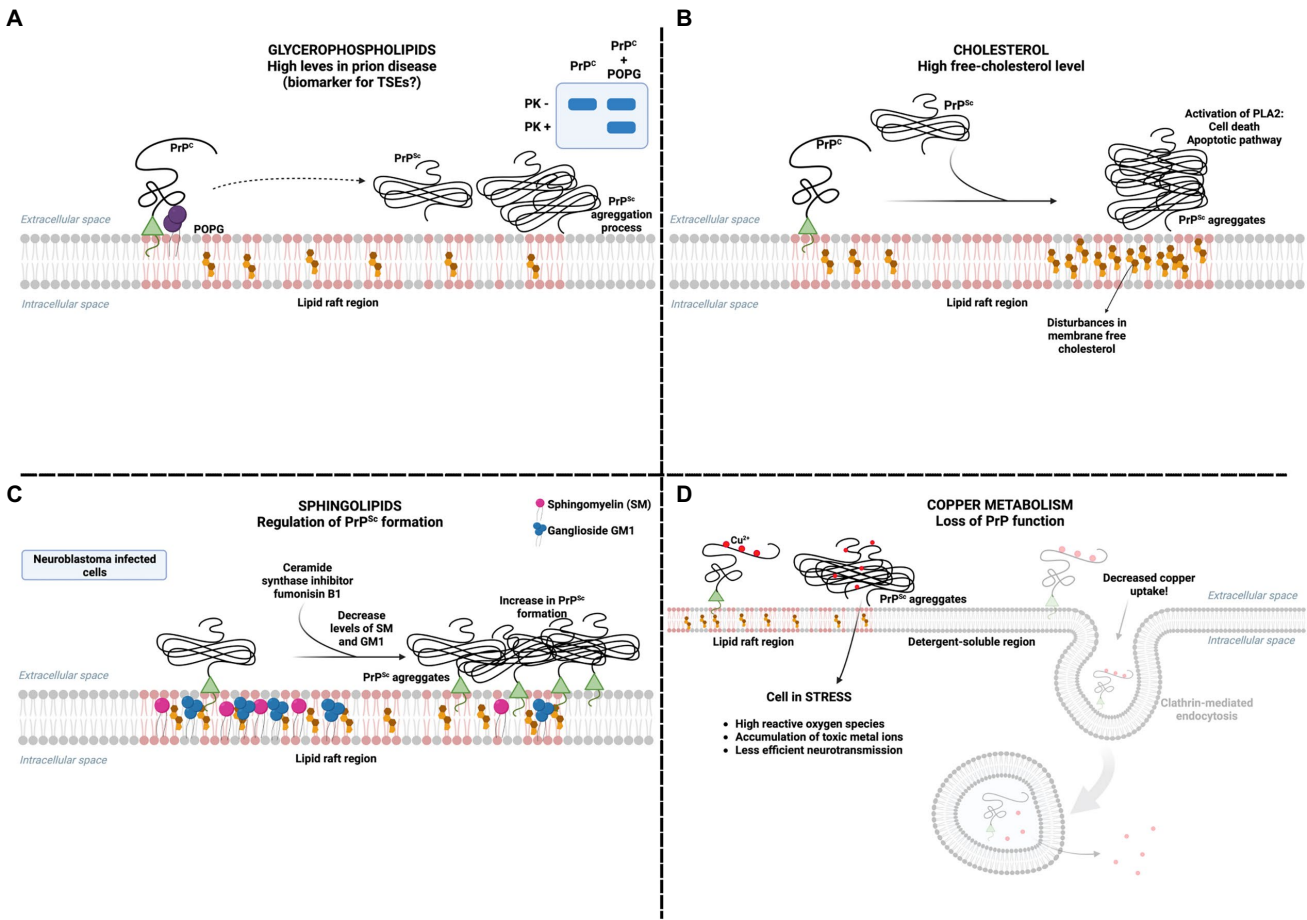
PrP^C^ interaction with lipids in pathology **(A)** Glycerophospholipids are proposed as potential biomarkers for TSEs as their concentration increases in prion disease models. The interaction of PrP with POPG can modulate the formation of proteinase K-resistant PrP^Sc^ aggregates. **(B)** It has already been reported that the presence of PrP^Sc^ increases free cholesterol and activates cell death mechanisms by activating phospholipase A (PLA). **(C)** The presence of sphingolipids in the membrane is essential in inhibiting the presence of PrP^Sc^. Depletion of sphingolipids SM and GM1, using the ceramide synthase inhibitor fumonisin B(1), has been reported to increase the presence of PrP^Sc^ in neuroblastoma cells infected with PrP^Sc^. **(D)** The presence of PrP^Sc^ in the plasma membrane prevents the internalization of copper, promoting a series of harmful consequences to the cell, mainly related to oxidative stress. Created with BioRender.com.

The pathophysiology of prion infection may be related to PrP^C^ loss of function or PrP^Sc^ gain of cytotoxic function (Winklhofer et al., 2008). A well-established characteristic of PrP^C^ is its N-terminal octapeptide repeat region binding affinity for Cu^2+^, which is required for some of the PrP^C^ physiological roles. PrP^C^ binding to Cu^2+^ regulates Cu^2+^ levels in neuronal cells, playing a neuroprotective role. Thus, PrP^C^ conversion to PrP^Sc^ causes perturbations in neuronal antioxidant activity (Figure 2D).

Cu^2+^ may be directly involved with PrP conversion and transmission. Cu^2+^ binding to octarepeats leads to PrP conformational change to a beta-sheet-rich structure (Salzano et al., 2019). Chelation of Cu^2+^ delayed prion disease in infected mice model (Sigurdsson et al., 2003). The presence of extra octapeptide regions (Krasemann et al., 1995) or the deletion of this region (Flechsig et al., 2000) impacts the formation of PrP^Sc^, being favored when this region is available, probably binding Cu^2+^. On the other hand, Cu^2+^ was also beneficial for prion diseases once its supplementation prolonged survival time in an infected animal model and protected N2A cells from infection (Hijazi et al., 2003). Once it enhances PrP^C^ internalization, it should reduce the encounter with PrP^Sc^ and, consequently, its conversion. The controversial results observed for Cu^2+^ must be further investigated for complete understanding. The findings probably result from a delicate balance between Cu^2+^ and PrP metabolism and its beneficial and deleterious roles.

### 3.2. Phospholipid-induced prion conversion

Two molecular hypotheses for PrP conversion have been investigated in recent decades. The protein-only hypotheses suggest that the presence of PrP^Sc^ alone can induce PrP^C^ structural changes and conversion (Prusiner, 1998; Baskakov and Bocharova, 2005). However, the energy barrier between both PrP structures could not be transposed only by the influence of PrP^Sc^ (Cohen and Prusiner, 1998; Silva et al., 2010b). Therefore, other macromolecules co-purified with PrP^Sc^ from brain tissues were suspected to be involved in PrP conversion. One of the main findings was that polyanionic compounds, such as RNA and proteoglycans, interact and convert endogenous and bacterially expressed PrP^C^ (recombinant PrP-rPrP; Deleault et al., 2003, 2007; Kovachev et al., 2019).

Following the finding that RNA was an essential molecule for PrP conversion, and since the RNA molecule is negatively charged and PrP is found at lipid membranes, it was suggested that negatively charged lipids could interact and induce PrP conversion (Table 1). PMCA (protein misfolding cyclic amplification) is a methodology that uses recombinant PrP as substrate and infected brain homogenates as seed. Preparations containing supposedly converting molecules can also be used in place of PrP^Sc^ seeds. The sample goes through sonication cycles, and PK-resistant PrP formation is observed if you have conversion and aggregation. Using this technique, Wang et al. (2010a) showed that both RNA and synthetic palmitoyl oleoyl-phosphoglycerol (POPG) caused recombinant PrP conversion to a PK-resistant form capable of propagating its conformation to endogenous PrP^C^ and causing clinical signs of prion disease when inoculated in wild-type mice. POPG vesicles induced the exposure of PrP-RNA binding sites leading to RNA direct interaction and PrP aggregation (Miller et al., 2013; Zurawel et al., 2014). In contrast, another study suggested that POPG and RNA may refold PrP^C^ to its PK-resistant form, PrP^Sc^-like, but lacking infectivity (Timmes et al., 2013). This contrasting result is probably a consequence of the depletion of cofactors necessary for maintaining PrP^Sc^ infectious conformation (Gomes et al., 2008; Deleault et al., 2012a,b). In addition, spectroscopic methods showed that POPG vesicles alone induce recombinant PrP conversion to a β-sheet enriched form, resistant to PK digestion (Wang et al., 2007; Sanghera et al., 2011), suggesting that membrane lipids could interact and convert PrP in the absence of any other molecule.

**Table 1:** Described effects of phospholipids with different charges on prion protein (PrP).

Phospholipid	Charge	Effect on PrP	References
POPG	Negative	Structural alterations	Morillas et al. (1999)
POPC	Zwitterion	No interaction	Morillas et al. (1999)
POPG	Negative	Structural alterations	Kazlauskaite et al. (2003)
POPC	Zwitterion	Structural alterations	Kazlauskaite et al. (2003)
PA/PI/PS	Negative	PrP aggregation	Tsiroulnikov et al. (2009)
PE	Zwitterion	PrP aggregation	Tsiroulnikov et al. (2009)
Several	Several	No effect in the absence of RNA	Deleault et al. (2012a,b)
POPG	Negative	RNA induced conversion	Miller et al. (2013)
PE	Zwitterion	PrP^Sc^ propagation in the absence of RNA	Noble et al. (2015)
PE/POPG	Zwitterion/Negative	POPG interacts with PrP inducing conversion, but PE does not interact	Srivastava and Baskakov (2015)
PA	Negative	Interact and induce aggregation	Angelli et al. (2021)

PA, phosphatidic acid; PE, phosphatidylethanolamine; PI, phosphatidylinositol; POPC, palmitoyl oleoyl-phosphatidylcholine; POPG, palmitoyl oleoyl-phosphatidylglycerol; PS, phosphatidylserine.

**Figure 3:**
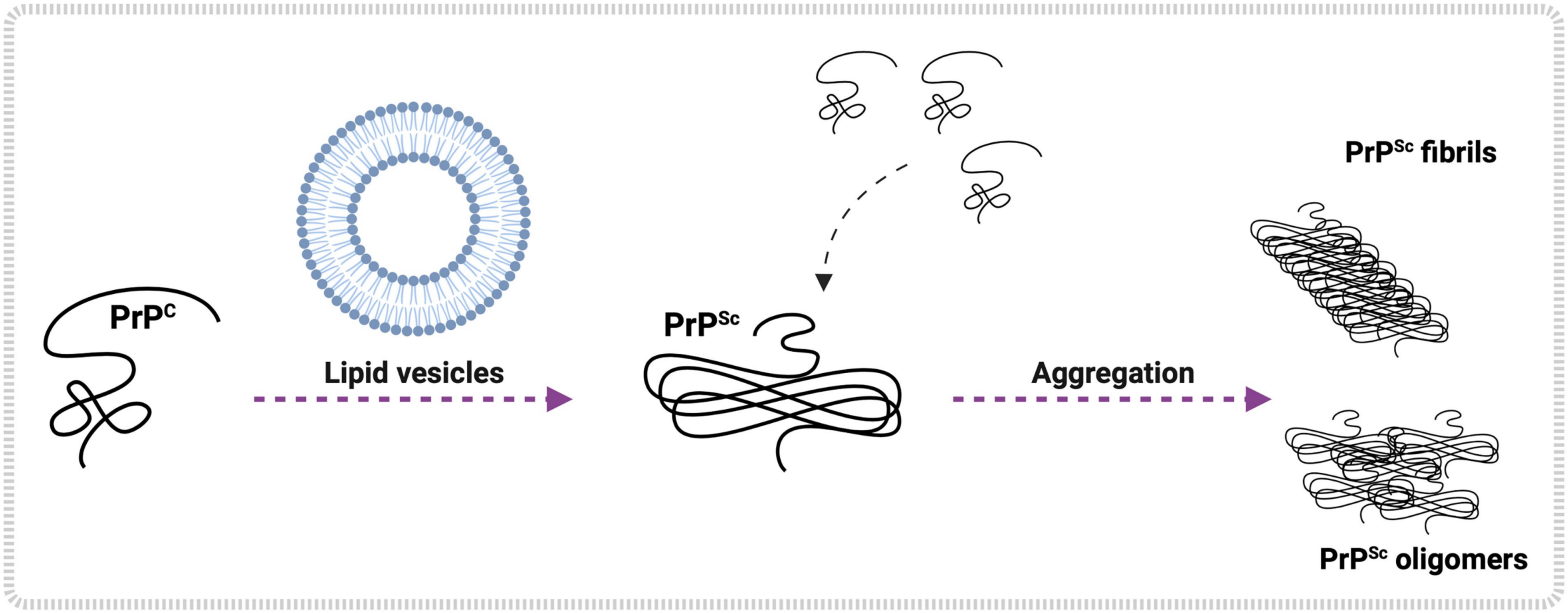
Lipid-mediated PrP^C^ conversion and aggregation *in vitro*. *In vitro* studies show lipid vesicle interaction with PrP^C^, leading to PrP^Sc^ formation. Examples of glycerophospholipids are POPG, PE, PA, and others. Once formed, PrP^Sc^ converts more PrP^C^ to the pathological form, initiating an aggregation process that may culminate in the shape of PrP^Sc^ oligomers and PrP^Sc^ fibrils. Created with BioRender.com.

Different negative and zwitterionic phospholipids can interact and induce PrP aggregation, such as PI, PS, PA, PE, and PC (Tsiroulnikov et al., 2009; Table 1 and Figure 3). Studies suggest that the interaction of phospholipids with PrP is linked to the charge of the lipid and other types of interactions, like van der Waals and hydrophobic forces, and may be determinants for phospholipid-induced PrP aggregation. PA vesicles were shown to interact and induce aggregation of either murine (MuPrP) and rabbit PrP (RbPrP), with greater affinity for RbPrP, despite leading to a more significant aggregation of MuPrP (Angelli et al., 2021). The higher affinity of RbPrP for PA is probably due to its more positively charged surface (Wen et al., 2010). Positively charged residues 100-110 at the PrP N-terminus and the hydrophobic region were necessary for PrP-POPG vesicle interaction, where the positively charged residues would be responsible for the first contact with POPG by electrostatic interactions. Together with the hydrophobic region (residues 111-134), these residues were critical for POPG-induced PrP conversion (Wang et al., 2010b).

It is still unclear how PrP-lipid interactions occur, and more studies are needed to explain the pathways in which these interactions may take place. Contrasting results from the literature suggest that differences in sample preparation, lipid systems, and methodologies can generate different effects on PrP conversion and aggregation. Deleault et al. (2012a) isolated PE as a lipid cofactor capable of inducing PrP conversion to infectious PrP without any other molecules. In contrast, Srivastava and Baskakov (2015) showed that PE did not significantly alter PrP^C^ structure nor lead to aggregation. Both studies worked with very similar buffer conditions but employed different lipid systems and methodologies. Deleault et al. (2012a) dissolved lipid powder in Triton and evaluated conversion after PMCA cycles. Srivastava and Baskakov (2015) prepared PE vesicles and evaluated the direct interaction with rPrP through different biophysical techniques without employing any amplification procedure.

Another study by Hoover et al. (2017) dissolved lipid powder in a chloroform-methanol solvent and performed the RT-QuIC (real-time quaking-induced conversion) assay. RT-QuIC, like PMCA, is an amplification assay that uses recombinant PrP as substrate and brain homogenates as seeds. Still, in specific buffer conditions, agitation cycles under temperatures around 55°C allow conversion only by PrP^Sc^ seeds. This work evaluated the effect of PI, PC, and PE and showed that these lipids did not induce PrP conversion when used as seed. When added together with Chronic wasting disease brain homogenates, it inhibited prion-seeded amyloid formation, suggesting an inhibitory effect for these lipids.

When comparing results from different cell models, different recombinant proteins used *in vitro*, and different lipid preparations and methodologies, it is difficult to define specific and clear roles for lipid cofactors, resulting in paradoxical results. At the same time, various approaches explore different characteristics of the PrP-lipid system, generating significant contributions. More studies on this topic will be essential to reveal the different paths that this interaction can take.

### 3.3. Mechanisms of lipid-mediated toxicity

Although the interaction of PrP with membrane lipids determines its function in cell physiology, in models of prion pathology, lipids have also been identified as mediators of prion toxicity. PrP aggregation involves the formation of oligomers, which are thought to be the neurotoxic forms rather than monomers or fibrils, with hydrophobic regions that may insert into lipid membranes and lead to destabilization (Simoneau et al., 2007). Since interactions of PrP with membrane lipids, such as POPG, were believed to induce a conformational change of PrP^C^ to a PrP^Sc^-like structure (Wang et al., 2007; Figure 2A), lipids emerged as potential cofactors in the formation of PrP neurotoxic aggregates in TSEs.

The infection of neuronal cell lines with PrP^Sc^ leads to an alteration in cell membrane composition, increasing free-cholesterol levels (Figure 2B), an effect not generated by PrP^C^ or by increasing overall cholesterol synthesis (Bate et al., 2008a,b,c), suggesting that the disturbance on membrane composition culminating in high levels of free cholesterol may be part of the mechanism of neurotoxicity in prion pathologies. Indeed, cholesterol was shown to stabilize prion multimers and may be required for the efficient formation of PrP^Sc^ (Taraboulos et al., 1995). Also, inhibiting the esterification of cholesterol by acyl-coenzyme A:cholesterol acyltransferase in neuronal cell lines was significantly more toxic for prion-infected cells (Bate et al., 2008a,b,c), suggesting that esterification of free cholesterol may be an important protective mechanism against PrP neurotoxicity. Inhibiting cholesterol synthesis protected primary neurons from cell death induced by platelet-activating factor (PAF; Bate et al., 2007), a phospholipid implicated in neuronal damage in different brain diseases, including TSEs. Therefore, there is strong evidence for cholesterol’s role as a mediator of PrP toxicity.

The apparent importance of the localization of PrP on lipid rafts during the conversion of PrP^C^ to PrP^Sc^ (Taylor and Hooper, 2006; Wälzlein et al., 2021) may be related to lipid-mediated toxicity in prion diseases. Indeed, the composition of the bilayer membrane may be a determinant of the PrP oligomer’s cytotoxicity. As in a mimetic model of the bacterial anionic membrane, oligomers led to membrane disruption through the detergent model (as proposed for other antimicrobial peptides). In contrast, in a model of the mammalian cell membrane, which is zwitterionic and contains cholesterol-rich domains, it induced a loss of domain separation and has been associated with the activation of apoptotic pathways (Walsh et al., 2014).

Two polyunsaturated fatty acids, docosahexaenoic and eicosapentaenoic acids, even with lower cholesterol levels, increased PrP^C^ expression and PrP^Sc^ formation in prion-infected neuronal cell lines. This was followed by increased activation of cytoplasmic phospholipase A2 (Bate et al., 2008a,b,c), whose inhibition was shown to prevent prion-induced neuronal damage (Last et al., 2012). These studies suggest that fatty acids may also mediate the toxicity of PrP aggregates.

Depletion of sphingolipids in prion-infected neuroblastoma cells, using the ceramide synthase inhibitor fumonisin B(1), led to a 4-fold increase in PrP^Sc^ formation, which seemed to inversely correlate specifically with sphingomyelin levels (Naslavsky et al., 1999; Figure 2C). Keeping in mind the importance of sphingolipid-rich rafts in the formation of PrP^Sc^ and the possible relation with its cytotoxicity (Taylor and Hooper, 2006; Walsh et al., 2014), alterations in the levels of membrane sphingolipids may be involved in neuronal damage in TSEs.

To date, the precise way PrP^Sc^ causes neurodegeneration is still unclear; studies suggest that it is related either to the deposition of PrP^Sc^ fibrils on cellular membranes, to PrP^C^ loss of function after its conversion, or to the conversion process itself (Westergard et al., 2007). Moreover, the clinical signs of prion disorders appear only in the late stages of the disease, hindering the possibility of early diagnosis (Connor et al., 2019). This, in turn, hinders the development of effective therapeutic strategies for blocking prion diseases. Although many polymers capable of impairing PrP^Sc^ accumulation or diminishing the incubation time of TSEs in neuronal cells have been developed in the last decades, none of them has been effective in humans (Teruya and Doh-ura, 2022). Interestingly, in a mouse model of prion disease, global changes in lipidomic profiling were demonstrated in the disease early stage; 75% of the alterations were on GLPs, upregulated in prion-infected animals, suggesting GLPs as potential lipid biomarkers for TSEs (Kim et al., 2021). Although more studies are needed, lipid alterations may allow an early diagnosis of prion diseases before clinical signs appear, representing a significant therapeutical potential in TSEs.

## 4. Concluding remarks

Over the last decade, many studies have revealed mechanistic similarities between prion diseases and other diseases that involve protein aggregation. For this reason, these proteins have been termed prion-like or prionoids (Ritchie and Barria, 2021). The sporadic nature of these diseases reveals the intricate role of factors that can be altered throughout the turnover of these proteins to favor the establishment of pathological pathways. Protein interaction with macromolecules is a factor in this process (Silva et al., 2010a; Burke et al., 2020); as such, their interaction with membranes and their lipid repertoire are relevant.

In this review, we showed the relationship between lipid membranes and PrP physiology, highlighting its importance in copper metabolism, remedying cellular stress conditions, and contributing to cell viability and migration. We also address the importance of PrP-lipid interaction in PrP^Sc^ conversion and pathology. Since PrP is a GPI-anchored membrane protein, it traffics from its synthesis to its recycling and degradation, facing different lipid repertoires. These different environments directly interfere with its stability and its propensity for conversion. Many phospholipids interact with PrP and recapitulate their conversion to toxic and infectious aggregates *in vitro*. PrP^Sc^ interaction with lipid membranes is also responsible for neuronal damage.

While *in vitro* models are vital for understanding details of more complex processes at the cellular and organismal level, recapitulating the complexity of the cellular environment, especially of the plasma membrane, is a significant challenge. Most studies have used vesicle models to assess the specific importance of certain lipids and to understand the physicochemical details of these interactions (Wang et al., 2007; Sanghera et al., 2011; Angelli et al., 2021). However, these lipid systems are poor in membrane composition and organization regarding lipids and the other molecules that compose these structures. Nanodiscs isolated from cell membranes using SMALPs (styrene maleic acid lipid particles) show lipid and protein profiles biologically relevant (Overduin et al., 2021). SMALPs can fragment membranes maintaining protein and lipid integrity. Studies with native nanodiscs are increasing and may bring important information about new membrane complex structures, improving the field of structural membrane biology. It will enhance the knowledge about the prion environment and partners, which is important for understanding physiology and pathology.

Understanding the role of lipids in prion physiology and pathology will lead to the development of therapeutic agents for prion diseases. Cerebral cholesterol originates most from *de novo* synthesis (Dai et al., 2021). So, modulation of its metabolism is an attractive therapeutic approach. Interestingly, prion-infected neurons show increased unesterified cholesterol levels due to the up-regulation of cholesterol synthesis enzymes (Bach et al., 2009) and inhibition of cholesterol export (Sodero, 2021). Some studies about the administration of cholesterol synthesis inhibitors, such as simvastatin and pravastatin, showed increased survival and delayed clinical signs in animal models (Kempster et al., 2007; Haviv et al., 2008; Vetrugno et al., 2009), but another study found no effect (Carroll et al., 2017). These findings demonstrate the need to carry out well-controlled experiments to exclude experimental and analysis variables among the research carried out and for more studies to understand the real benefit of these strategies better.

The imbalance of cholesterol levels occurs together with changes in sphingolipid and glycerophospholipid metabolism (Kim et al., 2021), including increased levels of PE that showed to be an *in vitro* conversion cofactor (Deleault et al., 2012a). Changes observed in lipid profile in the early stage of the disease in infected animal models (Kim et al., 2021) suggest that they are consequences of the infection but do not exclude the possibility that they are also involved with the cause of the disease. Targeting the conversion process and understanding the effect of lipids through direct conversion or modulation of PrP localization can be interesting therapeutically.

PrP^Sc^ propagates through four main mechanisms: direct cell-to-cell contact, tunneling nanotubes, GPI painting (spontaneous incorporation of GPI-protein into the cell surface membrane), and extracellular vesicles (Heumüller et al., 2022). Alterations of the lipid membrane profile may interfere with many of these processes: (i) Changes in GPI anchor or even detachment of PrP from the cell membrane will directly interfere with cell-to-cell propagation and GPI painting. (ii) Changes in cholesterol and sphingolipids perturb lipid raft domains and PrP localization, also interfering with cell-to-cell propagation and GPI painting. (iii) Modulating the endocytosis process will affect the intercellular transmission of endocytic compartments through tunneling nanotubes and extracellular vesicles. All these approaches are exciting to be therapeutically investigated.

Although we know a lot about the importance of lipids for prion diseases, many questions remain unanswered about their role in the conversion and propagation of PrP^Sc^. The extent to which changes in the cellular lipid profile are a cause or consequence of the conversion and propagation of PrP remains to be elucidated. The aggregation pathways involved in the interaction with lipids also need to be explained to understand better the molecular mechanism involved in establishing prion diseases, thus enabling the development of relevant therapeutic strategies. The mechanisms are still quite elusive, bringing an exciting perspective for developing new studies in this area. Since the membrane environment is essential for the aggregation and toxicity of other prion-like proteins, the findings relating to PrP are of considerable importance for prion diseases and many other diseases.

## Author contributions

TV and CA contributed to the conception and design of the study. TV, CA, GL, and CB wrote sections of the manuscript. All authors contributed to the manuscript revision and read and approved the submitted version.

## Funding

This study was financed in part by the Coordenação de Aperfeiçoamento de Pessoal de Nível Superior – Brasil (CAPES) – Finance Code 001; Fundação de Amparo à Pesquisa do Estado do Rio de Janeiro (FAPERJ); and the Conselho Nacional de Desenvolvimento Científico e Tecnológico (CNPq).

## Conflict of interest

The authors declare that the research was conducted in the absence of any commercial or financial relationships that could be construed as a potential conflict of interest.

## Publisher’s note

All claims expressed in this article are solely those of the authors and do not necessarily represent those of their affiliated organizations, or those of the publisher, the editors and the reviewers. Any product that may be evaluated in this article, or claim that may be made by its manufacturer, is not guaranteed or endorsed by the publisher.
